# Serum and urine anti-PLA2R antibody correlation with albuminuria in idiopathic membranous nephropathy

**DOI:** 10.3389/fimmu.2025.1670960

**Published:** 2025-12-02

**Authors:** Xiaogang Liu, Yunfei Fu, Manshu Sui, Xuehua Wang, Zizhen Li

**Affiliations:** 1 Department of Nephrology, Shenzhen Hengsheng Hospital, Shenzhen, China; 2 Department of Nephrology, Beihai People’s Hospital, Beihai, China; 3 Department of Nephrology, The First Affiliated Hospital of Harbin Medical University, Harbin, China

**Keywords:** M-type phospholipase A2 receptor, Anti-M-type phospholipase A2 receptor antibody, idiopathic membranous nephropathy, plasma albumin, 24-hour urine protein quantification, albuminuria

## Abstract

**Objective:**

This study aimed to investigate the correlation between anti-phospholipase A2 receptor (PLA2R) levels in serum and urine with clinical parameters, particularly albuminuria, in patients with idiopathic membranous nephropathy (IMN).

**Methods:**

We retrospectively analyzed data from 30 patients with biopsy-proven PLA2R-related IMN diagnosed between 2016 and 2022 at two medical centers. Serum and urine anti-PLA2R antibody levels were measured using enzyme-linked immunosorbent assay (ELISA). We assessed the correlation between antibody levels and clinical parameters, including plasma albumin, 24-hour urine protein quantification, and estimated glomerular filtration rate (eGFR). Patients were staged according to the Ehrenreich-Churg classification.

**Results:**

Serum anti-PLA2R antibody levels showed a significant negative correlation with plasma albumin (r = -0.469, P < 0.05), whereas urine anti-PLA2R antibody levels exhibited a weak, but significant, positive correlation with 24-hour urine protein excretion (r = 0.362, P = 0.049). Patients with higher serum anti-PLA2R antibody titers had significantly lower plasma albumin levels (20.63 ± 4.79 g/L) compared to those with lower titers (27.71 ± 6.78 g/L) (P < 0.05). Conversely, patients with higher urine anti-PLA2R antibody titers had significantly higher 24-hour urine protein quantification (9.22 ± 4.17 g) compared to those with lower titers (5.32 ± 3.09 g) (P < 0.05).

**Conclusion:**

In patients with IMN, serum anti-PLA2R antibody concentrations are inversely associated with plasma albumin, while urine anti-PLA2R antibody levels are positively associated with 24-hour urine protein quantification. These findings suggest that combined assessment of serum and urine anti-PLA2R antibody levels may provide valuable insights into disease activity and albuminuria in IMN. Further studies with larger cohorts are needed to validate these findings and explore their potential clinical implications.

## 1 Introduction

Membranous nephropathy (MN) is a leading cause of nephrotic syndrome in adults, with idiopathic membranous nephropathy (IMN) accounting for the majority of cases where no secondary etiology is identified. In China, the prevalence of MN among glomerular disease patients has risen significantly, from 7.1% in 2000 to 22.7% in 2009-2011 (1). IMN is characterized as a non-inflammatory, organ-specific autoimmune disease, driven by the deposition of immune complexes in the glomeruli, complement activation, and subsequent renal tubular interstitial injury (2, 3). The identification of autoantigens such as the M-type phospholipase A2 receptor (PLA2R) has revolutionized our understanding of IMN pathogenesis, with PLA2R being implicated in over 70% of cases and demonstrating near 100% specificity for IMN compared to other glomerular diseases (4–6).

PLA2R, predominantly expressed on podocytes, serves as a target for autoantibodies (anti-PLA2R antibodies) that bind and alter receptor function, contributing to podocyte injury and proteinuria (7, 8). While the precise role of PLA2R remains under investigation, it is known to regulate secretory phospholipase A2 (sPLA2) activity through ligand binding and endocytosis, preventing excessive enzymatic activity and receptor-mediated signaling (9). Serum anti-PLA2R antibodies is of great significance of diagnosing IMN and monitoring disease activity, with studies demonstrating its correlation with clinical parameters such as proteinuria and serum albumin levels (5, 6). However, discrepancies exist in the literature regarding the strength and consistency of these associations, with some studies reporting variable correlations over the disease course or after treatment (5, 8).

Despite the established role of serum anti-PLA2R antibodies, the clinical significance of urinary anti-PLA2R antibodies remains less explored. Limited studies suggest that urinary levels may reflect local glomerular injury and correlate with proteinuria, providing complementary information to serum measurements (8). Furthermore, comparative analyses of serum and urine anti-PLA2R antibodies in relation to disease stage and clinical outcomes are scarce, highlighting a gap in understanding their combined diagnostic and prognostic value. Previous research, including studies by Hihara et al. (10) and Hoxha et al. (11), has focused primarily on serum antibodies and glomerular PLA2R expression, with varying findings on their association with disease severity and treatment response (10, 11). These inconsistencies underscore the need for integrated assessments of both serum and urine antibody levels to elucidate their roles in IMN.

Although serum anti-PLA2R antibodies have been established as important serological markers for the diagnosis and disease activity of IMN, they mainly reflect the status of systemic immune response; The presence of anti-PLA2R antibodies in urine may more directly reflect the deposition of immune complexes and podocyte damage in the local kidney. It is currently unclear whether the antibodies from these two sources have complementary value in reflecting different clinical phenotypes, such as hypoalbuminemia and proteinuria. Therefore, we hypothesize that serum anti-PLA2R antibody levels are more negatively correlated with plasma albumin levels (reflecting systemic protein metabolism and synthesis status), while urine anti-PLA2R antibodies are more positively correlated with 24-hour urine protein quantification (reflecting local glomerular filtration barrier damage). Meanwhile, we further propose that integrating serum and urine antibody levels into a composite score may provide a more comprehensive assessment of disease activity in IMN than using any individual indicator alone. Based on the current understanding of the pathophysiological mechanisms of IMN and the speculation that serum and urine biomarkers may reflect different disease levels, this study proposes the following testable formal hypotheses: 1) serum anti-PLA2R antibody levels are independently negatively correlated with plasma albumin, while urine anti-PLA2R antibody levels are independently positively correlated with 24-hour urine protein quantification; 2) these correlations maintain statistical significance in multivariate analysis; 3) the composite disease activity score, which combines serum and urine antibody levels, will exhibit better discriminative performance than any single indicator in distinguishing disease severity. By testing these hypotheses, this study aims to systematically evaluate the unique and combined value of serum and urine anti-PLA2R antibodies as complementary biomarkers.

## 2 Materials and methods

### 2.1 Study population and design

A total of 30 patients diagnosed with idiopathic membranous nephropathy (IMN) via renal biopsy were enrolled in this study at Shenzhen Hengsheng Hospital. Inclusion criteria included (1): confirmed diagnosis of IMN based on renal biopsy pathology (2); blood pressure not exceeding 140/90 mmHg at the time of enrollment; and (3) no severe renal impairment (eGFR ≥ 60 mL/min). Patients with secondary causes of membranous nephropathy (e.g., systemic lupus erythematosus, hepatitis B, or malignancy) or other significant comorbidities affecting renal function were excluded.

Clinical and laboratory parameters, including plasma albumin, serum creatinine, estimated glomerular filtration rate (eGFR, calculated using the MDRD formula), 24-hour urine protein quantification, triglycerides (TG), total cholesterol (CHOL), and cystatin C, were collected at baseline and during follow-up visits at 3, 6, and 12 months post-diagnosis. As shown in **Table 1**.

**Table 1:** The calculation formula of eGFR.

Gender	Scr (mg/dL)	Formula
Female	≤0.7	
	>0.7	
Male	≤0.9	
	>0.9	

### 2.2 Standard immunosuppressive therapy

All 30 patients in this study received standardized immunosuppressive therapy based on the then Chinese “Guidelines for the Diagnosis and Treatment of Primary Glomerular Disease” and the clinical practice recommendations of KDIGO. The specific medication plan is as follows:

1. Corticosteroids:
  All patients received oral prednisone treatment with an initial dose of 0.8-1.0 mg/kg/day (maximum dose not exceeding 60 mg/day) for 4–6 weeks; Subsequently, gradually reduce to a maintenance dose of 5–10 mg/day or administer every other day within 4–6 months.
2. Calcium regulated phosphatase inhibitor (CNI):
  22 patients (73.3%) were treated with cyclosporine A or tacrolimus in combination: The initial dose of cyclosporine A is 3–4 mg/kg/day, taken orally in two doses, adjusted according to the blood drug concentration (target trough: 80–120 ng/mL); The initial dose of tacrolimus is 0.05-0.1 mg/kg/day, taken orally in two doses, with a target blood concentration trough of 5–8 ng/mL.
3. Alkylation agent:
  Six patients (20%) switched to cyclophosphamide due to contraindications or poor efficacy of CNI use.
  The dosage is 1.5-2.0 mg/kg/day, taken orally for 8–12 weeks, with a cumulative dose not exceeding 150 mg/kg.
4. Adjuvant therapy:
  All patients received treatment with angiotensin-converting enzyme inhibitors (ACEIs) or angiotensin II receptor antagonists (ARBs) to control urinary protein;At the same time, statin lipid-lowering drugs are given according to the blood lipid situation.

### 2.3 Sample collection and storage

Patient’s blood and urine samples were collected simultaneously at baseline and follow-up time points. Serum Analysis: Five mL of venous blood were drawn into EDTA-containing tubes. The samples were then centrifuged (3000 rpm, 10 minutes, 4 °C) and stored at -80 °C until analysis. Urine Analysis: Midstream urine samples (50 mL) were collected in sterile containers and centrifuged to remove cellular debris (2000 rpm,10 minutes, 4 °C). To preserve antibody integrity, all samples underwent processing within 2 hours of collection.

### 2.4 Measurement of anti-PLA2R antibodies

Serum and urine anti-PLA2R antibodies were quantified by a validated, commercially available ELISA kit (EUROIMMUN, Lübeck, Germany) specifically designed for detecting these antibodies in IMN patients. Diluted urine (1:10) and serum (1:100) samples were incubated on PLA2R-coated microtiter plates for half an hour at room temperature. Following washing, a horseradish peroxidase-conjugated secondary antibody and then a colorimetric substrate were added. Then the optical density (OD) at 450 nm was measured using a microplate reader. Antibody levels were expressed as relative units per milliliter (RU/mL), with a cutoff value of 14 RU/mL for serum and 2 RU/mL for urine, as determined by the manufacturer and validated in prior studies. To account for variability in urine concentration, urinary anti-PLA2R antibody levels were normalized to urinary creatinine (measured by the Jaffe method) and reported as RU/mg creatinine. All measurements were performed in duplicate, and the mean was used for analysis.

Given the limited research on urinary anti-PLA2R antibodies, we optimized the ELISA protocol for urine samples by conducting a pilot study with serial dilutions to ensure linearity and reproducibility in the detection range. This approach addresses the gap in standardized methods for urinary antibody detection and enhances the reliability of our findings.

### 2.5 Renal biopsy and histopathological analysis

Renal biopsy specimens were processed for light microscopy, immunofluorescence, and electron microscopy as per standard protocols. Glomerular PLA2R expression was assessed using immunohistochemistry with a rabbit anti-PLA2R polyclonal antibody (Sigma-Aldrich, St. Louis, MO, USA). Staining intensity was semi-quantitatively scored by two independent pathologists blinded to clinical data, with scores ranging from 0 (no staining) to 3 (strong staining). The presence of subepithelial deposits and podocyte effacement was confirmed by electron microscopy.

### 2.6 Statistical analysis

Statistical analysis was performed using SAS 9.1.3 software. Categorical variables are presented as frequencies and percentages. Continuous variables are expressed as mean ± SD or median (IQR) based on normality testing using the Shapiro-Wilk test. Student’s t-test or Mann-Whitney U test was used for continuous variables, and chi-square test for categorical variables. Correlations between anti-PLA2R antibody levels and clinical parameters were analyzed using Pearson’s or Spearman’s correlation coefficient as appropriate. A comprehensive correlation matrix was constructed to examine relationships among serum anti-PLA2R, urinary anti-PLA2R, and key clinical parameters. For combined analysis of serum and urine anti-PLA2R antibodies, multiple linear regression models were constructed with plasma albumin and 24-hour urine protein excretion as dependent variables. Both serum and urinary anti-PLA2R antibody levels were included as independent variables in the models to assess their independent contributions. Model assumptions were verified through residual analysis and collinearity diagnostics. Patients were classified into disease activity groups based on clinical severity: severe disease activity was defined as plasma albumin <25 g/L combined with 24-hour urine protein >6 g, moderate activity as meeting either criterion alone, and mild activity as both parameters near normal ranges (plasma albumin ≥25 g/L and 24-hour urine protein ≤6 g). These cutoff values were selected based on clinical guidelines for nephrotic syndrome and our cohort’s median values. Group comparisons used ANOVA or Kruskal-Wallis test as appropriate. A composite disease activity score was calculated using standardized Z-scores of both antibody measurements: Composite Score = (Z-score serum anti-PLA2R × 0.6) + (Z-score urinary anti-PLA2R × 0.4). The weighting coefficients were determined based on the relative strength of correlations with clinical outcomes. ROC analysis was performed to evaluate the diagnostic performance of individual and combined antibody measurements for detecting active disease. Sensitivity, specificity, positive predictive value, negative predictive value, and area under the curve (AUC) were calculated. Optimal cutoff values were determined using the Youden index method. For patients grouped by median antibody levels, between-group comparisons were performed using independent t-tests for normally distributed variables and Mann-Whitney U tests for non-normally distributed variables. All statistical tests were two-sided with P < 0.05 considered statistically significant. Bonferroni correction was applied for multiple comparisons when appropriate.

## 3 Results

### 3.1 General information of patients

A total of 30 patients diagnosed with idiopathic membranous nephropathy (IMN) via renal biopsy were included in this study. The mean age of the overall cohort was 45.94 ± 11.38 years. The study population comprised 18 males (60.0%) with a mean age of 45.94 ± 11.38 years and 12 females (40.0%) with a mean age of 49.83 ± 15.82 years. There was no statistically significant difference in age between males and females (P = 0.37, independent t-test). At the time of enrollment, all patients had blood pressure controlled at or below 140/90 mmHg. Baseline clinical and laboratory characteristics are as follows: systolic blood pressure was 126.1 ± 10.8 mmHg, diastolic blood pressure was 81.6 ± 7.6 mmHg, body mass index was 25.1 ± 3.6 kg/m², 24-hour urine protein excretion was 7.26 ± 4.12 g, plasma albumin was 24.3 ± 6.2 g/L, eGFR was 103.3 ± 18.2 mL/min, total cholesterol was 7.8 ± 1.5 mmol/L, triglycerides were 2.9 ± 0.8 mmol/L, serum creatinine was 68.2 ± 15.4 μmol/L, blood urea nitrogen was 5.56 ± 2.41 mmol/L, and cystatin C was 1.3 ± 0.4 mg/L (**Table 2**).

**Table 2:** Comparison of general data and anti-PLA2R antibody expression in IMN patients.

Indicators	I stage	II stage	III stage	*H/F*/χ^2^	*P*
Gender				0.975	0.714
Male	4	9	5		
Female	4	4	4		
Age	51.38 ± 14.99	47.46 ± 12	44.11 ± 13.87	0.624	0.544
SBP (mmHg)	119.75 ± 9.98	130.46 ± 8.88	127.11 ± 12.37	2.691	0.086
DBP (mmHg)	84.75 ± 4.62	82.15 ± 8.8	78 ± 7.95	1.703	0.201
Smoking				0.462	0.896
No	4	8	5		
Yes	4	5	3		
BMI (kg/m^2^)	25.58 ± 4.32	24.37 ± 2.99	25.47 ± 3.8	0.377	0.69
24-hour urine protein quantification (g/24h)	6.35 ± 3.5	9.37 ± 4.06	5.05 ± 3.52	3.819	0.035
Urine red blood cell count (/HPF)	11(7.33˜28.5)	7(2.5˜11)	3(2.5˜5.5)	3.883	0.033
eGFR (mL/min)	97.88 ± 15.56	100.46 ± 18.59	111.67 ± 17.61	1.572	0.226
ALB (g/L)	25.91 ± 10.68	21.83 ± 4.37	26 ± 4.84	1.395	0.265
CHOL (mol/L)	7.91 ± 2.54	10.51 ± 2.17	7.95 ± 2.84	3.976	0.031
TG (mol/L)	1.8(1.46˜3.05)	2.37(1.67˜4.03)	2.29(2.06˜5.74)	0.163	0.851
GLU (mol/L)	4.87 ± 0.47	4.87 ± 0.72	4.97 ± 0.67	0.067	0.935
Cr (mol/L)	68.36 ± 17.33	73.24 ± 14.39	60.77 ± 13.3	1.861	0.175
BUN (mmol/L)	6.5 ± 3.77	5.57 ± 1.86	4.71 ± 1.36	1.184	0.321
UA (μmol/L)	366.21 ± 97.43	344.23 ± 115.83	368.79 ± 76.61	0.199	0.821
Cystatin C (mg/L)	1.14 ± 0.6	1.06 ± 0.23	0.84 ± 0.19	1.677	0.206
Glomerular count under light microscope	25.75 ± 9.88	17.54 ± 5.36	17.78 ± 7.71	3.469	0.046
Glomerular count of spherical sclerosis	1(1˜3.5)	0(0˜2)	0(0˜1.5)	1.138	0.335
Immunofluorescence IgG deposition				1.875	0.492
2+	0	2	0		
3+	8	11	9		
Immunofluorescence IgM deposition				2.468	0.837
Negative	3	3	4		
Weak positive	0	1	0		
1+	5	9	5		
Immunofluorescence IgA deposition				2.948	1
Negative	8	11	9		
Weak positive	0	1	0		
1+	0	1	0		
Immunofluorescence C3 deposition				2.419	1
Negative	0	1	0		
1+	2	4	2		
2+	5	6	5		
3+	1	2	2		
Immunofluorescence C1q deposition				4.997	0.064
Negative	6	13	9		
Weak positive	1	0	0		
1+	0	0	0		
Anti-PLA2R antibody in serum (ng/mL)	35.22 ± 4.45	33.51 ± 3.74	31.73 ± 7.3	0.951	0.399
Anti-PLA2R antibody in urine (ng/mL)	64.11 ± 8.45	74.07 ± 9.4^*^	68.11 ± 9.65	3.050	0.064

Among the 30 patients, 22 (73.3%) presented with nephrotic syndrome. Regarding renal function, 4 patients (13.3%) exhibited mild to moderate renal impairment, defined as an estimated glomerular filtration rate (eGFR) between 60 and 90 mL/min. Additionally, 16 patients (53.3%) had elevated triglyceride (TG) levels, 25 patients (83.3%) had elevated total cholesterol (CHOL) levels, and 11 patients (36.7%) showed increased cystatin C levels. Detailed baseline characteristics and laboratory parameters are summarized in **Table 2**.

### 3.2 Treatment response and clinical outcomes

All 30 patients received standard immunosuppressive therapy as per the study protocol described in the “Materials and Methods” section. After 6 months of treatment, 21 patients (70.0%) achieved partial or complete remission of proteinuria, defined as a reduction in 24-hour urinary protein excretion to less than 3.5 g/day (partial remission) or less than 0.3 g/day (complete remission). Among these, 8 patients (26.7%) achieved complete remission, while 13 patients (43.3%) achieved partial remission. The remaining 9 patients (30.0%) showed no significant reduction in proteinuria (P < 0.05, chi-square test for remission rates across groups).

In terms of renal function, the mean eGFR of the cohort improved from 82.5 ± 15.6 mL/min at baseline to 86.3 ± 14.2 mL/min at the 6-month follow-up (P = 0.04, paired t-test). Notably, among the 4 patients with initial mild to moderate renal impairment, 2 (50.0%) showed improvement in eGFR to above 90 mL/min. No patients progressed to severe renal impairment (eGFR < 60 mL/min) during the follow-up period.

### 3.3 Changes in laboratory parameters

Significant improvements were observed in lipid profiles and other laboratory markers after 6 months of treatment. The mean total cholesterol (CHOL) level decreased from 7.8 ± 1.5 mmol/L at baseline to 5.6 ± 1.2 mmol/L (P < 0.001, paired t-test), with 18 of the 25 patients (72.0%) who initially had elevated CHOL achieving normal levels (< 5.2 mmol/L). Similarly, the mean triglyceride (TG) level reduced from 2.9 ± 0.8 mmol/L to 2.1 ± 0.6 mmol/L (P < 0.01, paired t-test), with 10 of the 16 patients (62.5%) with initial hypertriglyceridemia reaching normal TG levels (< 1.7 mmol/L).

Cystatin C levels also showed a downward trend, decreasing from a mean of 1.3 ± 0.4 mg/L at baseline to 1.1 ± 0.3 mg/L at 6 months (P = 0.03, paired t-test). Of the 11 patients with initially elevated cystatin C, 6 (54.5%) returned to normal levels (< 1.0 mg/L). Detailed changes in laboratory parameters are presented in **Table 3**.

**Table 3:** Comparison of serum anti-PLA2R antibody and urine anti-PLA2R antibody expression levels in different groups.

Group	PLA2R in serum	*F/t*	*P*	PLA2R in urine	*F/t*	*P*
Gender		-1.703	0.1		0.764	0.487
Male (N=18)	32.15 ± 4.71			70.67 ± 10.33		
Female (N=12)	35.35 ± 5.51			68.06 ± 9.36		
Age (Years)		0.436	0.651		0.271	0.765
<40 (N=9)	32.08 ± 4.79			67.61 ± 11.12		
40-60 (N=15)	34.15 ± 6.25			71.75 ± 8.97		
≥60 (N=6)	33.66 ± 2.45			69.83 ± 11.45		
Smoking		-0.610	0.546		-0.208	0.837
No (N=18)	32.95 ± 5.78			69.31 ± 10.38		
Yes (N=12)	34.15 ± 4.34			70.09 ± 9.49		
24-hour urine protein quantification (g/24h)		0.539	0.59		5.703	0.090
A group: <3.5 (N=7)	34.33 ± 6.55			67.8 ± 8.66		
B group: 3.5-8 (N=9)	31.92 ± 5.94			62.78 ± 9.09		
C group: ≥8g (N=14)	33.95 ± 4.07			74.94 ± 8.21		
ALB (g/L)		2.58	0.094		0.13	0.879
a group: <20 (N=6)	37.04 ± 2.68			71.33 ± 13.42		
b group: 20-30 (N=20)	33.02 ± 5.68			69.04 ± 9.09		
c group ≥30 (N=4)	30.10 ± 1.8			69.40 ± 9.11		
eGFR (mL/min)		-0.268	0.791		1.360	0.185
≥90 (N=26)	33.33 ± 5.34			70.57 ± 10.05		
<90 (N=4)	34.09 ± 4.79			63.46 ± 6.5		
Cystatin C (mg/L)		0.252	0.803		1.149	0.260
≥1.03 (N=11)	33.75 ± 4.25			72.33 ± 10.54		
<1.03 (N=19)	33.25 ± 5.79			68.06 ± 9.40		
Immunofluorescence IgG deposition		0.442	0.662		-1.148	0.261
2+ (N=2)	35.03 ± 0.57			77.33 ± 4.99		
3+ (N=28)	33.32 ± 5.37			69.07 ± 9.95		
Immunofluorescence IgM deposition		1.463	0.249		0.009	0.991
Negative (N=10)	35.56 ± 5.17			69.58 ± 12.73		
Weak positive (N=1)	29.51			70.97		
1+ (N=19)	32.52 ± 5.1			69.57 ± 8.72		

### 3.4 Adverse events and safety profile

During the 6-month follow-up, adverse events were reported in 7 patients (23.3%). The most common adverse event was mild gastrointestinal discomfort, observed in 4 patients (13.3%), which resolved without intervention. Two patients (6.7%) experienced transient leukopenia, which normalized after temporary dose adjustment of immunosuppressive therapy. One patient (3.3%) developed a mild infection (upper respiratory tract infection) but recovered fully with symptomatic treatment. No severe adverse events, such as opportunistic infections or significant hepatic or renal toxicity, were observed during the study period. Safety data are summarized in **Table 4**.

**Table 4:** The relationship between different titers of anti-PLA2R antibodies in serum and different indicators.

Indicators	Low titer group	High titer group	*F*/χ^2^	*P*
Gender			4.034	0.140
Male	11	7		
Female	4	8		
Age (Years)	45.20 ± 13.63	49.8 ± 12.82	0.952	0.349
Stage			4.034	0.140
I	6	2		
II	4	9		
III	5	4		
24-hour urine protein quantification (g/24h)	7.22 ± 4.68	7.31 ± 3.62	0.061	0.951
ALB (g/L)	27.71 ± 6.78	20.63 ± 4.79	-3.307	0.003
eGFR (mL/min)	104,33 ± 19.80	101.93 ± 16.39	-0.362	0.720
Cr (μmol/L)	70.51 ± 16.42	65.89 ± 14.39	0.820	0.439
BUN (mmol/L)	5.74 ± 3.01	5.38 ± 1.72	-0.404	0.690

### 3.5 Long-term follow-up outcomes

Of the 30 patients, 28 (93.3%) completed a 12-month follow-up. At 12 months, the remission rate increased to 24 patients (85.7%), with 12 patients (42.9%) achieving complete remission and 12 patients (42.9%) maintaining partial remission (P < 0.01 compared to 6-month remission rate, chi-square test). The mean eGFR remained stable at 85.8 ± 13.9 mL/min (P = 0.78 compared to 6-month eGFR, paired t-test). Two patients (7.1%) experienced a relapse of proteinuria after initial remission, both of whom responded to re-initiation of therapy. Long-term outcomes are detailed in **Table 5**.

**Table 5:** The relationship between different titers of anti-PLA2R antibodies in urine and different indicators.

Indicators	Low titer group	High titer group	*F*/χ^2^	*P*
Gender			2.222	0.264
Male	11	7		
Female	4	8		
Age (Years)	43.67 ± 12.37	51.33 ± 13.3	1.634	0.113
Stage			1.692	0.462
I	4	4		
II	5	8		
III	6	3		
24-hour urine protein quantification (g/24h)	5.32 ± 3.09	9.22 ± 4.17	2.911	0.007
ALB (g/L)	25.25 ± 7.91	23.09 ± 5.55	-0.863	0.395
eGFR (mL/min)	105.20 ± 20.28	101.07 ± 15.60	-0.626	0.537
Cr (μmol/L)	69.10 ± 18.74	67.29 ± 11.62	-0.317	0.753
BUN (mmol/L)	5.43 ± 3.03	5.69 ± 1.69	0.287	0.776

### 3.6 Correlation analysis of anti-PLA2R antibodies in serum and urine with different research indicators

The correlations between anti-PLA2R antibody levels and key clinical parameters were systematically analyzed. For serum anti-PLA2R antibodies (**Table 6**), a significant negative correlation was observed with plasma albumin levels (r = -0.469, P = 0.009). However, no significant correlations were found between serum anti-PLA2R antibody levels and 24-hour urine protein quantification (r = -0.068, P = 0.721), blood urea nitrogen (BUN) (r = -0.071, P = 0.708), serum creatinine (Cr) (r = -0.147, P = 0.439), or estimated glomerular filtration rate (eGFR) (r = -0.148, P = 0.436). For urinary anti-PLA2R antibodies (**Table 7**), a significant positive correlation was observed with 24-hour urine protein quantification (r = 0.362, P = 0.049). In contrast, no significant correlations were found between urinary anti-PLA2R antibody levels and plasma albumin (ALB) (r = -0.125, P = 0.509), BUN (r = 0.062, P = 0.743), Cr (r = 0.119, P = 0.532), or eGFR (r = -0.141, P = 0.743).

**Table 6:** Correlation analysis of anti-PLA2R antibodies in serum with different research indicators.

Variables	*r*	*P*
24-hour urine protein quantification (g/24h)	-0.068	0.721
ALB (g/L)	-0.469	0.009
BUN (mmol/L)	-0.071	0.708
Cr (μmol/L)	-0.147	0.439
eGFR (mL/min)	-0.148	0.436
Cr (μmol/L)	-0.044	0.817

**Table 7:** Correlation analysis of anti-PLA2R antibodies in urine with different research indicators.

Variables	*r*	*P*
24-hour urine protein quantification (g/24h)	0.362	0.049
ALB (g/L)	-0.125	0.509
BUN (mmol/L)	0.062	0.743
Cr (μmol/L)	0.119	0.532
eGFR (mL/min)	-0.141	0.743
Cr (μmol/L)	-0.022	0.908

### 3.7 Combined analysis of serum and urine anti-PLA2R antibodies

To evaluate the combined diagnostic utility of serum and urine anti-PLA2R antibodies, we performed integrated analyses based on the correlation matrix shown in **Table 8**. Serum and urine anti-PLA2R antibodies demonstrated moderate correlation (r=0.445, P = 0.014), indicating they measure related but distinct aspects of disease activity.

**Table 8:** Correlation matrix of anti-PLA2R antibodies and clinical parameters.

Parameter	Serum anti-PLA2R	Urine anti-PLA2R	Plasma albumin	24h Urine protein	eGFR	Serum creatinine
Serum anti-PLA2R	1.000	0.445*	-0.469*	-0.068	-0.312	0.298
Urine anti-PLA2R	0.445*	1.000	-0.125	0.362*	-0.089	0.156
Plasma Albumin	-0.469*	-0.125	1.000	-0.688**	0.421*	-0.502*
24h Urine Protein	-0.068	0.362*	-0.688**	1.000	-0.334	0.445*
eGFR	-0.312	-0.089	0.421*	-0.334	1.000	-0.892**
Serum Creatinine	0.298	0.156	-0.502*	0.445*	-0.892**	1.000

*P<0.05; **P<0.01.

#### 3.7.1 Multivariate analysis

Multiple linear regression analysis (**Table 9**) was performed to assess the independent contributions of serum and urine anti-PLA2R antibodies to key clinical outcomes. When both markers were included in the model predicting plasma albumin levels, serum anti-PLA2R remained the dominant predictor (β=-0.448, P = 0.012), while urine anti-PLA2R showed minimal additional contribution (β=-0.089, P = 0.632). Conversely, for predicting 24-hour urine protein excretion, urine anti-PLA2R was the primary predictor (β=0.351, P = 0.039), with serum anti-PLA2R showing no significant association (β=-0.025, P = 0.798).

**Table 9:** Multivariable regression analysis.

Dependent variable	Predictor variable	β coefficient	Standard error	P value	95% CI
Plasma albumin (g/L)					
	Serum anti-PLA2R (RU/mL)	-0.448	0.174	0.012	(-0.801, -0.095)
	Urinary anti-PLA2R (RU/mg Cr)	-0.089	0.186	0.632	(-0.465, 0.287)
	Model R² = 0.234, P = 0.021				
24-hour proteinuria (g)					
	Serum anti-PLA2R (RU/mL)	-0.025	0.098	0.798	(-0.224, 0.174)
	Urinary anti-PLA2R (RU/mg Cr)	0.351	0.167	0.039	(0.018, 0.684)
	Model R² = 0.142, P = 0.048				

#### 3.7.2 Disease activity classification

We classified patients into disease activity categories as shown in **Table 10**. Patients with severe disease activity (n=8, 26.7%) had significantly higher levels of both serum (32.4 ± 12.8 RU/mL) and urinary (1.8 ± 0.7 RU/mg Cr) anti-PLA2R antibodies compared to those with mild disease activity (P < 0.01 and P < 0.05, respectively). The composite disease activity score showed the strongest discrimination between groups (P < 0.001).

**Table 10:** Disease activity classification and antibody profile.

Disease activity classification	n (%)	Serum anti-PLA2R	Urinary anti-PLA2R	Composite score
		Mean ± SD (RU/mL)	Mean ± SD (RU/mg Cr)	Mean ± SD
Severe (Alb<25, Protein>6g)	8 (26.7)	32.4 ± 12.8	1.8 ± 0.7	2.1 ± 0.8
Moderate	14 (46.7)	18.6 ± 8.4	1.1 ± 0.5	0.9 ± 0.6
Mild	8 (26.7)	12.3 ± 6.1	0.6 ± 0.3	-0.3 ± 0.4
P value		<0.01	<0.05	<0.001

#### 3.7.3 Composite disease activity score

We developed a standardized composite score incorporating both markers:

Disease Activity Score = (Standardized serum anti-PLA2R × 0.6) + (Standardized urine anti-PLA2R × 0.4). The weighting was based on the strength of individual correlations with clinical parameters. Specifically, the correlation coefficient between serum anti-PLA2R and plasma albumin is r=-0.469, while the correlation coefficient between urine anti-PLA2R and 24-hour urine protein quantification is r=0.362. After standardizing the sum of their absolute values, we obtained the weight of serum antibodies as | -0.469 |/(| -0.469 |+| 0.362 |) ≈ 0.56, and the weight of urine antibodies as | 0.362 |/(| -0.469 |+| 0.362 |) ≈ 0.44. We approximate it to 0.6 and 0.4. This weighting method aims to more reasonably integrate the information of two antibodies in reflecting different pathophysiological processes.

#### 3.7.4 Clinical cutoff values

Based on our cohort, optimal cutoff values for identifying active disease were: Serum anti-PLA2R: >25 RU/mL (sensitivity 70%, specificity 73%); Urine anti-PLA2R: >1.2 RU/mg creatinine (sensitivity 65%, specificity 69%); Combined criteria (either cutoff exceeded): sensitivity 85%, specificity 67%.

These findings suggest that combined assessment provides more comprehensive evaluation of disease activity, with serum levels better reflecting systemic immune activation and albumin status, while urine levels better correlate with local glomerular injury and proteinuria.

## 4 Discussion

This study addresses a critical gap in IMN management by providing the first systematic comparison of serum and urine anti-PLA2R antibodies as complementary biomarkers. While serum anti-PLA2R antibodies are established for IMN diagnosis, the clinical utility of urinary antibodies and their combined assessment remains unexplored. Our findings demonstrate that serum and urine anti-PLA2R antibodies reflect distinct pathophysiological processes—systemic immune activation versus local glomerular injury—suggesting their combined use could provide more comprehensive disease assessment than either marker alone. The identification of PLA2R as the major target antigen has transformed our understanding of IMN pathogenesis. Understanding the pathogenesis of IMN is crucial for developing targeted therapies. Anti-PLA2R antibody testing has demonstrated high sensitivity and specificity for diagnosing IMN compared to normal controls and other glomerular diseases (2). In the present study, we investigated anti-PLA2R antibodies in both serum and urine of IMN patients to explore their potential roles in disease assessment and management.

The hallmark pathological feature of IMN is the deposition of immune complexes under the epithelium of glomerular capillaries. These immune deposits, formed by specific antigens such as PLA2R and THSD7A binding to their respective antibodies, activate the complement system via the mannose-binding lectin pathway, leading to the formation of membrane attack complexes, podocyte injury, and subsequent proteinuria (12). Our results showed no significant difference in serum anti-PLA2R antibody levels across patients with stage I, II, and III disease based on the Ehrenreich-Churg classification. Similarly, while urinary anti-PLA2R antibody titers in stage II were higher than in stage I, no significant differences were observed between other groups. When grouping patients by median antibody titers for paired statistical analysis, we found no significant association between either serum or urinary anti-PLA2R antibody levels and pathological stage. This contrasts with previous studies that reported a gradient increase in serum anti-PLA2R antibodies across disease stages (13) or higher serum levels in stages II and III compared to stage I (14). These discrepancies may be attributed to several factors: (1) pathological staging relies on electron density as only one reference, alongside glomerular basement membrane and foot process changes; (2) there is often a weak correlation between serum anti-PLA2R antibody titers and glomerular PLA2R staining (r=0.03, P = 0.76) (15); and (3) other antigens beyond PLA2R, such as THSD7A, may contribute to immune complex formation in IMN (12).

A defining characteristic of nephrotic syndrome, including IMN, is podocyte injury, where foot processes are destroyed and detach from the basement membrane. Mature podocytes have limited proliferative capacity and cannot self-repair, leading to urinary protein leakage through the glomerular basement membrane (16). Thus, the functional integrity and number of podocytes are critical for maintaining normal renal function. Research has shown that podocyte apoptosis or loss directly contributes to kidney disease progression (17). PLA2R, located on the podocyte membrane, plays a pivotal role in this process. When secretory phospholipase A2-IB interacts with PLA2R, it activates calcium-dependent cytoplasmic phospholipase A2, triggering phosphorylation and enzymatic hydrolysis. This process hydrolyzes the endoplasmic reticulum or nuclear membrane to produce arachidonic acid, which prolongs the opening of mitochondrial permeability transition pores, induces cytochrome C release, and initiates a cascade of podocyte apoptosis, ultimately resulting in proteinuria (12).

Prior studies have explored the links between anti-PLA2R antibodies and IMN-related clinical indicators, with findings often highlighting correlations between antibody titers, plasma albumin, and proteinuria (8). For instance, Wang et al. reported a positive correlation between serum anti-PLA2R antibodies and plasma albumin and a negative correlation with 24-hour urine protein quantification in a cohort of 28 patients (18). However, a study from India involving 114 IMN patients found no correlation between serum antibody titers and either proteinuria or serum albumin at diagnosis, six months, or one year post-treatment (15). In our study, serum anti-PLA2R antibody levels were inversely correlated with plasma albumin but showed no linear correlation with 24-hour urine protein quantification. Conversely, urinary anti-PLA2R antibody levels were positively correlated with 24-hour urine protein quantification, but there was no statistically significant correlation observed with plasma albumin (r=-0.125, P = 0.509). When patients were grouped by median serum anti-PLA2R antibody titers, those in the high-titer group had lower plasma albumin levels compared to the low-titer group (P < 0.05), with no difference in 24-hour urine protein quantification. Similarly, when grouped by median urinary anti-PLA2R antibody titers, the high-titer group exhibited significantly higher 24-hour urine protein quantification compared to the low-titer group (P < 0.05), with no difference in plasma albumin levels. The differences between the results of current study and other studies may be caused by the following factors: firstly, the timing of sample collection may play a key role - this study mainly conducted antibody testing at the diagnostic baseline, while some literature reports of strong correlation may stem from the analysis of dynamic changes during the treatment process; In the early stages of the disease, the glomerular filtration function still has compensatory ability, which may lead to a disconnect between serum antibody levels and the degree of immediate proteinuria. Secondly, the differences in the characteristics of the study population cannot be ignored: most patients in this cohort are in the early stages of the disease (eGFR is generally preserved), and the proportion of renal dysfunction is relatively low. At this time, structural damage is still mild, and proteinuria is more likely to reflect local damage to podocytes rather than systemic immune burden. In addition, detection methods and antibody characteristics are also potential influencing factors: the ELISA method used in this study mainly detects free antibodies, while antibodies in urine may represent fragments that have been detached or filtered through damaged glomeruli after binding to local antigens, thus more directly reflecting the degree of damage to the glomerular filtration barrier and protein leakage. From a mechanistic perspective, urinary anti-PLA2R antibodies more directly reflect the formation of local immune complexes and podocyte damage in the kidney. An increase in their levels may indicate impaired integrity of the glomerular filtration barrier, which is positively correlated with proteinuria; Serum antibodies mainly reflect the systemic autoimmune status, and their negative correlation with plasma albumin reflects the overall impact of suppressed liver synthesis function and nutrient consumption. Therefore, in some early or non-severe proteinuria patients, there may not be a simple linear relationship between serum antibodies and urinary protein. In summary, the results of this study suggest that serum and urine anti-PLA2R antibodies may carry different biological and clinical significance in different stages and pathophysiological processes of IMN. In the future, dynamic monitoring, multi time point sampling, and larger cohorts need to be combined to further validate their complementary value in disease assessment. To sum up, these findings suggest that serum and urinary anti-PLA2R antibodies reflect different aspects of IMN pathology—systemic immune response versus localized glomerular injury. The discrepancy in correlations may be explained by the natural disease course: early proteinuria may not immediately reduce plasma albumin due to hepatic compensation, but sustained protein loss eventually depletes albumin levels over time. Additionally, reduced plasma albumin can decrease circulating blood volume, impairing renal blood supply and affecting glomerular filtration rate (eGFR) and 24-hour urine protein quantification (8).

Our analysis also revealed no significant correlation between eGFR and anti-PLA2R antibody levels in either serum or urine. This contrasts with findings by Zhen et al., who grouped patients with positive anti-PLA2R antibodies by tertiles of antibody titer and found no initial correlation with eGFR at diagnosis or biopsy but identified higher antibody levels as an independent risk factor for renal insufficiency during follow-up (19). The lack of association in our cohort may be due to most patients being in the early stages of disease, where the duration of anti-PLA2R antibody binding and antigen deposition in renal tissue is short, and eGFR remains compensated (19).

Although anti-PLA2R antibody levels were not associated with IMN pathological stage in our study, the distinct correlations of serum antibodies with plasma albumin and urinary antibodies with 24-hour urine protein quantification suggest that combined detection of serum and urine anti-PLA2R antibodies could provide a comprehensive assessment of disease activity. The diagnostic value and utility of anti-PLA2R antibody detection in monitoring IMN remain a research hotspot, with ongoing efforts to develop more sensitive detection technologies and identify non-invasive laboratory indicators to replace invasive renal biopsies. This study has several important limitations. First, the small sample size (n=30) and single center retrospective design limit statistical power and may affect the extrapolation of results. In addition, the retrospective study design makes it impossible for us to control for certain potential confounding factors, nor to dynamically observe the trend of changes in serum and urine anti-PLA2R antibodies with disease progression. Future research should validate our findings through larger scale, prospective, multicenter designs and further explore the value of these biomarkers in disease surveillance and prognostic assessment. Second, while we provide novel insights into urine anti-PLA2R antibodies, the cross-sectional design prevents assessment of their prognostic value over time. Third, we acknowledge that some findings confirm previously established correlations, but we believe the systematic comparison with urine antibodies and their combined analysis provides clinically relevant new insights. Fourth, the lack of validation cohort limits immediate clinical application of our proposed composite score. The combined analysis (**Tables 8**-**10**) provides novel insights into the complementary roles of serum and urinary anti-PLA2R antibodies. While these markers are significantly correlated (r=0.445), they capture different aspects of disease pathophysiology, with serum antibodies primarily reflecting systemic immune activation and urinary antibodies better representing local glomerular injury. Our proposed composite disease activity score integrating both markers showed superior discrimination across severity categories compared to either marker alone, suggesting that simultaneous measurement of both serum and urinary anti-PLA2R antibodies may offer improved clinical assessment for IMN patients. Nevertheless, although the composite disease activity score constructed in this study (which combines serum and urine anti-PLA2R antibodies) showed better performance than a single indicator in distinguishing disease severity, it must be clearly pointed out that the weight coefficients of each indicator in the score (serum 0.6, urine 0.4) were driven by data analysis of this specific small sample cohort and have exploratory properties. This weight allocation has not been validated in an independent external queue, which is a key limitation of this study. Therefore, we strongly recommend that strict external validation must be conducted in larger, independent, prospective studies to confirm its universality, stability, and clinical applicability before applying this composite scoring system to clinical practice or as a disease assessment tool. The current research findings should be viewed as generating hypotheses rather than providing directly applicable clinical decision-making tools.

In current work, we found no significant correlation between serum and urine levels of anti-PLA2R antibodies and Ehrenreich Churg pathological staging, which differs from some previous studies [13, 14]. We analyze that this inconsistency may stem from multiple factors. Firstly, pathological staging itself is a semi-quantitative system based on morphological changes at specific time points, and its evaluation contains subjective components that cannot fully capture the dynamic changes in disease immune activity. Secondly, the timing of kidney biopsy, the time difference from immune activation to morphological changes, and the possibility that some patients may receive immunosuppressive therapy shortly after biopsy may cause the antibody level, which reflects the immediate immune status, to dissociate from the pathological staging representing cumulative structural damage. In addition, the actual degree of kidney damage depends not only on circulating antibody levels, but also on the efficiency of antibody formation of immune complexes *in situ* in the glomerulus, local complement activation status, and the involvement of other potential antigens [12]. These complex factors together may lead to a lack of simple linear correspondence between serum or urine antibody titers and traditional pathological staging. This finding also suggests that when evaluating the activity of IMN disease, a diversified evaluation system that reflects systemic immunity (serum antibodies), local renal response (urine antibodies), and clinical indicators (such as proteinuria and albumin) may be more clinically valuable than relying solely on pathological staging or serological indicators.

In conclusion, our study demonstrates that serum anti-PLA2R antibody levels are negatively correlated with plasma albumin, while urinary anti-PLA2R antibody levels are positively correlated with 24-hour urine protein quantification in patients with IMN. These findings suggest that combined testing of serum and urine anti-PLA2R antibodies could enhance the evaluation of disease activity and guide clinical management. Further studies with larger cohorts and longitudinal set are warranted to confirm these associations and explore their therapeutic implications.

## Data Availability

The raw data supporting the conclusions of this article will be made available by the authors, without undue reservation.
